# Decitabine enhances targeting of AML cells by NY-ESO-1-specific TCR-T cells and promotes the maintenance of effector function and the memory phenotype

**DOI:** 10.1038/s41388-022-02455-y

**Published:** 2022-09-12

**Authors:** Synat Kang, Lixin Wang, Lu Xu, Ruiqi Wang, Qingzheng Kang, Xuefeng Gao, Li Yu

**Affiliations:** 1 Department of Hematology and Oncology, International Cancer Center, Shenzhen Key Laboratory of Precision Medicine for Hematological Malignancies, Shenzhen University General Hospital, Shenzhen University Clinical Medical Academy, Shenzhen University Health Science Center, Shenzhen, 518000 Guangdong China; 2 School of Medicine, Nankai University, Tianjin, 300071 China; 3 Central Laboratory, Shenzhen Key Laboratory of Precision Medicine for Hematological Malignancies, Shenzhen University General Hospital, Shenzhen, 518000 Guangdong, China

**Keywords:** Acute myeloid leukaemia, Diagnostic markers

## Abstract

NY-ESO-1 is a well-known cancer-testis antigen (CTA) with re-expression in numerous cancer types, but its expression is suppressed in myeloid leukemia cells. Patients with acute myeloid leukemia (AML) receiving decitabine (DAC) exhibit induced expression of NY-ESO-1 in blasts; thus, we investigated the effects of NY-ESO-1-specific TCR-engineered T (TCR-T) cells combined with DAC against AML. NY-ESO-1-specific TCR-T cells could efficiently eliminate AML cell lines (including U937, HL60, and Kasumi-1cells) and primary AML blasts in vitro by targeting the DAC-induced NY-ESO-1 expression. Moreover, the incubation of T cells with DAC during TCR transduction (designated as dTCR-T cells) could further enhance the anti-leukemia efficacy of TCR-T cells and increase the generation of memory-like phenotype. The combination of DAC with NY-ESO-1-specific dTCR-T cells showed a superior anti-tumor efficacy in vivo and prolonged the survival of an AML xenograft mouse model, with three out of five mice showing complete elimination of AML cells over 90 days. This outcome was correlated with enhanced expressions of IFN-γ and TNF-α, and an increased proportion of central memory T cells (CD45RO^+^CD62L^+^ and CD45RO^+^CCR7^+^). Taken together, these data provide preclinical evidence for the combined use of DAC and NY-ESO-1-specific dTCR-T cells for the treatment of AML.

## Introduction

Despite therapeutic advances in the treatment of acute myeloid leukemia (AML) in the past few years, the overall survival of AML patients is still poor due to primary and secondary resistance [1]. Allogeneic hematopoietic stem cell transplantation (allo-HSCT) can induce remission in AML patients through the T cell-mediated graft-versus-leukemia effect, but the success of this approach is limited, with relapse in approximately 30–50% of cases depending mainly on the disease status at the time of transplantation [2–4]. Furthermore, leukemia cells can also evade the immune system in AML patients after allo-HSCT via several mechanisms [5]. The efforts to reduce toxicity while preserving the anti-tumor efficacy of cytotoxic T cells, as well as to export this therapeutic opportunity beyond the allo-HSCT context, have driven the development of engineered cytotoxic T cells. The redirection of T cells with chimeric antigen receptor (CAR) and T cell receptor (TCR) has been reported to overcome the limitations of allo-HSCT in the treatment of relapsed and refractory leukemia [6–8]. CAR-T cells can recognize and kill tumor cells by binding target cell surface antigens in an MHC-unrestricted manner, which is the most common strategy for hematological treatment [9–11]. However, the absence of leukemia-specific surface antigens for CAR-T cells to target limits its application in leukemia immunotherapy [12]. Alternatively, TCR-transduced T-cells (TCR-T) can recognize intracellular antigens processed by major histocompatibility (MHC) proteins, which have revealed encouraging results in preclinical and clinical studies [13–15].

A variety of cancer-testis antigens (CTAs) have been recognized as potential targets for cancer immunotherapy, given their restricted expression in somatic tissues and aberrant expression in malignant cells [16, 17]. Among the CTA family, NY-ESO-1 is of particular interest, and the safety and efficacy of NY-ESO-1-specific immunotherapies have been demonstrated in a variety of tumors such as sarcoma, melanoma, myeloma, and non-small cell lung cancer [16, 18–21]. However, the expression of NY-ESO-1 and many other CTAs is suppressed in myeloid leukemia cells due to promoter hypermethylation [22–24], which is the main hurdle for immunotherapy for myeloid malignancies that target CTAs. Demethylating agents, such as 5-azacytidine (5AC) and 5-aza-2’-deoxycytidine (decitabine, DAC), can inhibit DNA methyltransferases, thereby leading to re-expression of tumor-suppressor genes, and are approved by the FDA to treat myelodysplastic syndromes (MDS) and AML. Our study and others have previously reported that DAC could induce the expression of CTAs, including NY-ESO-1, in circulating myeloid cells and leukemic blast in MDS/AML patients [23, 25–30]. Furthermore, the induction of NY-ESO-1 expression could activate a cytotoxic response from HLA-compatible NY-ESO-1-specific T lymphocytes [25], which has laid the basis for combining CTA-specific immunotherapy with demethylation agents for the treatment of AML. For example, NY-ESO-1 vaccination combined with DAC for the treatment of AML has established positive results in a phase I clinical study [27]. Although DAC-induced NY-ESO-1-specific cytotoxic T lymphocytes (CTLs) have been used in clinical settings, CTLs express wild-type TCRs, which have low affinity for their target under tumor-escaping conditions [31, 32].

In this study, we show that the combined use of DAC with high-affinity NY-ESO-1-specific TCR-T cells has a high efficacy against AML. Moreover, the treatment of TCR-T cells with DAC during transduction (designated as dTCR-T) can further enhance their anti-leukemia efficacy in vivo, and increase the memory phenotype with long-term persistence and anti-leukemia durability.

## Results

### Decitabine induces NY-ESO-1 expression in leukemia cell lines and primary AML blasts by demethylating the DNA promoter region

**Fig. 1:**
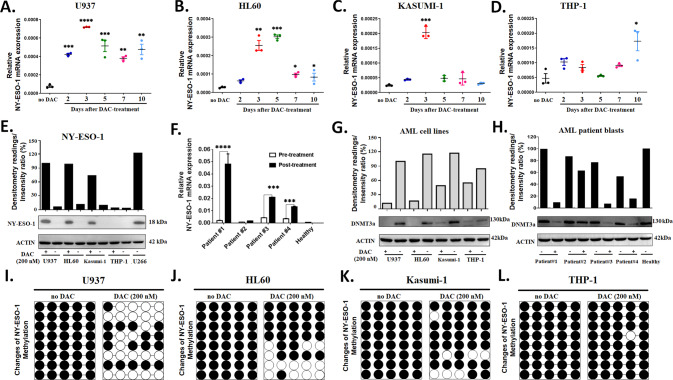
Decitabine induces NY-ESO-1 expression in AML cell lines through DNA demethylation. RT-PCR analysis of NY-ESO-1 levels in AML cell lines (**A**) U937, (**B**) HL60, (**C**) Kasumi-1, and (**D**) THP-1 at 10 days after treatment with 200 nM DAC. **E** Western blot analysis of the NY-ESO-1 protein level in AML lines before and 3 days after DAC treatment. The multiple myeloma cell line U266 was used as a positive control for NY-ESO-1 expression. **F** RT-PCR analysis of NY-ESO-1 mRNA levels in primary AML blasts before and after DAC treatment. The data in **A**–**D**, and **F** are presented as mean ± sd (*n* = 3). Two-tailed unpaired *t* tests were used to compare the pre-treatment with post-treatment groups. *P < 0.05; **P < 0.01; ***P < 0.001; ****P < 0.0001. Western blot analysis of the DNMT3a level in AML cell lines (**G**) and patient blast samples (**H**) before and after DAC treatment. The membranes were incubated with anti-DNMT3a, and β-actin was used as a loading control. Bisulfite sequencing analysis of the methylation status of NY-ESO-1 promoters in AML cell lines (**I**) U937, (**J**) HL60, (**K**) Kasumi-1, and (**L**) THP-1 treated with 200 nM DAC for 72 h (*n* = 8).

We treated four AML cell lines, including U937, HL60, Kasumi-1, and THP-1 cells, with various doses (100–1000 nM) of DAC for 72 h and observed an up-regulation of NY-ESO-1 in U937, HL60, and Kasumi-1 cell lines at both the mRNA and protein levels (Supplementary Fig. S1A–F). However, the expression of NY-ESO-1 did not change in THP-1 cells, suggesting a poor response to DAC (Supplementary Fig. S1G, H). By treating the AML cell lines with DAC at doses ranging from 100 nM to 1000 nM, we observed that the expression level of NY-ESO-1 peaked at 200 nM for U937, HL60, and Kasumi-1 cell lines. By monitoring the NY-ESO-1 expression for 10 days after treatment with 200 nM DAC, the highest level was detected at 3 days for U937, HL60, and Kasumi-1 cell lines, and the significantly high levels (relative to the untreated cells) were maintained until 10 days after exposure for U937 and HL60 cell lines (Fig. 1A–C). The THP-1 cell line showed only a moderate increase in the NY-ESO-1 mRNA level at 10 days of DAC treatment (Fig. 1D). The protein level of NY-ESO-1 after 3 days of DAC exposure was consistent with the results of the mRNA level (Fig. 1E). Thus, DAC induces NY-ESO-1 expression in AML cells in dose- and time-dependent manners.

Furthermore, we assessed NY-ESO-1 expression in primary blasts isolated from the bone marrow of four AML patients who were treated by DAC. Patient characteristics and chemotherapy regimens are presented in Table 1. A significant increase in the NY-ESO-1 mRNA level was detected in the primary blasts of three AML patients (patient nos. 1, 3, and 4; Fig. 1F), but not for patient no. 2 who had achieved a complete remission (<5% AML blasts in the bone marrow, blood cell counts are normal and absence of any disease signs or symptoms) after the chemotherapy. In addition, we accessed the safety of combined high-affinity TCR-T cells with DAC with human normal bone marrow and three normal cell lines (HK-2, proximal tubule epithelial cell; NCM460, colon mucosal epithelial cell; and MCF10A, breast epithelial cell), and found that the NY-ESO-1 expression was low in these normal cells and not affected by DAC treatment (Supplementary Fig. S2A–H). Thus, the effect of DAC on up-regulating NY-ESO-1 expression is restricted to AML cells.

DNA methyltransferase DNMT3a encodes an epigenetic regulator that mediates *de novo* methylation of CpG dinucleotides. We found that DNMT3a expression was significantly reduced by DAC treatment in U937, HL60, and Kasumi-1 cell lines, but not in the THP-1 cell line (Fig. 1G), indicating a differential susceptibility of AML subclones to DAC treatment among these cells. In the bone marrow samples, DNMT3a expression was significantly reduced in three AML patients (nos. 1, 3, 4) who showed no remission after DAC treatment, but not for patient no. 2 who achieved complete remission and a healthy donor (Fig. 1H). In addition, DAC did not affect DNMT3a expression in human normal bone marrow and normal cell lines (HK-2, NCM460, and MCF10A) (Supplementary Fig. S2M). Moreover, bisulfite sequencing analysis showed that DAC significantly induced the demethylation of the NY-ESO-1 promoter region in U937, HL60, and Kasumi-1 cell lines (Fig. 1I–K), but barely affected the methylation status of the NY-ESO-1 promoter in THP-1, HK-2, MCF10A, NCM460, and normal bone marrow cells (Fig. 1L**;** Supplementary Fig. S2I–L).

Taken together, these data reveal that the up-regulation of NY-ESO-1 in AML cells is associated with DAC-induced NY-ESO-1 promoter hypomethylation. Moreover, normal cells and the THP-1 cell line do not respond to the demethylation effect of DAC.

### Decitabine enhances NY-ESO-1-specific TCR-T cell-mediated recognition and killing of AML cells in vitro

Given the ability of DAC to induce NY-ESO-1 expression in AML cells, we investigated whether DAC could increase the recognition of AML cells by NY-ESO-1-specific TCR-T cells. TCR-expression in CD3^+^ and CD8^+^ cells confirmed a high transduction efficiency of NY-ESO-1-specific TCR-T (Supplementary Fig. S3).

**Fig. 2:**
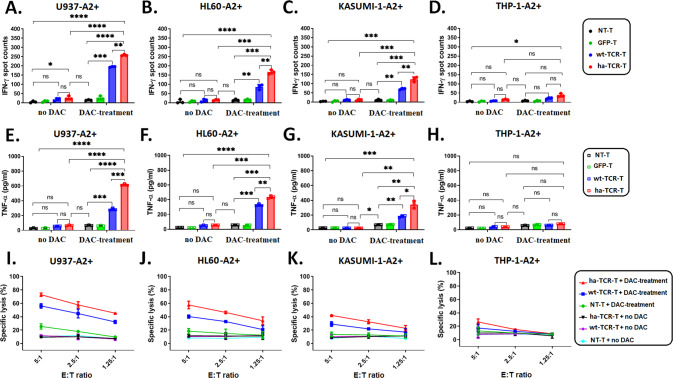
NY-ESO-1-specific high-affinity TCR-T cells kill AML cell lines by recognizing decitabine-induced NY-ESO-1. **A**–**D** ELISpot analysis of IFN-γ secretion by 2 × 10^3^ NT-T, GFP-T, wt-TCR-T, or ha-TCR-T cells were co-cultured with either untreated or DAC-treated target cells, including U937-A2^+^, HL60-A2^+^, Kasumi-1-A2^+^, and THP-1-A2^+^ cells at an effector-to-target (E:T) ratio of 1:10 for 20 h. **E**–**H** ELISA analysis of TNF-α expression by 1 × 10^5^ NT-T, GFP-T, wt-TCR-T, or ha-TCR-T cells that were stimulated by untreated or DAC-treated target cells at an E:T ratio of 5:1 for 20 h. The data in (**A**–**H**) are presented as mean ± sd (*n* = 3). Statistical comparisons between two groups were determined by two-tailed unpaired t tests. *P < 0.05; **P < 0.01; ***P < 0.001; ****P < 0.0001; *ns* not significant. **I**–**L** Specific lysis of target cells was measured by the LDH-release assay at different E:T ratios. The data are presented as mean ± sd (*n* = 3).

Our previous study demonstrated that high-affinity 1G4 TCR-T cells (K_D_ = 1.07 μM) showed enhanced killing activities than the wild-type 1G4 TCR-T cells (K_D_ = 32 μM) [32]. We found here, in consistence with our previous study, high-affinity TCR-T (ha-TCR-T; thereafter referred to as TCR-T in other sections) cells in combing with DAC remains better killing than the wild-type TCR-T (wt-TCR-T) cells against AML. The cytokine secretions and cytotoxicity were higher in ha-TCR-T than wt-TCR-T cells against U937-A2^+^, HL60-A2^+^, and Kasumi-1-A2^+^ cells, but not in THP-1-A2^+^ cells (Fig. 2A–L). IFN-γ and TNF-α were secreted by ha-TCR-T and wt-TCR-T cells co-cultured with DAC-treated U937-A2^+^, HL60-A2^+^, and Kasumi-1-A2^+^ cells, whereas low cytokine secretion was detected in the untreated controls. The level of cytokine secretion in response to the targets was higher in ha-TCR-T cells than wt-TCR-T cells (Fig. 2A–C, Fig. 2E–G). In addition, the ha-TCR-T and wt-TCR-T cells secreted higher levels of IFN-γ and TNF-α in response to DAC-treated U937-A2^+^ cells compared to those in response to DAC-treated HL60-A2^+^ and Kasumi-1-A2^+^ cells. Both ha- and wt-TCR-T cells cultured with THP-1-A2^+^ cells showed no activation, and with only minimal levels of cytokine secretion with or without DAC exposure (Fig. 2D, H). Thus, cytokine production by TCR-T cells is correlated with NY-ESO-1 expression by target cells.

The LDH assay was performed to evaluate the cytotoxicity of NY-ESO-1-specific ha-TCR-T and wt-TCR-T cells against AML cells with different E:T ratios (Fig. 2I–L). The untransduced T cells (NT-T), wt-TCR-T, and ha-TCR-T cells that were cultured with untreated target cells showed low cytolytic activity. Specific lysis of U937-A2^+^, HL60-A2^+^, and Kasumi-1-A2^+^ cells by NY-ESO-1-specific ha-TCR-T and wt-TCR-T cells were only observed when they were cultured with DAC (Fig. 2I–K). In addition, DAC did not trigger specific lysis of THP-1-A2^+^ cells by NY-ESO-1-specific the two TCR-T cells (Fig. 2L). Taken together, these data indicate that DAC can promote the recognition and killing of AML cells by NY-ESO-1-specific TCR-T cells in vitro.

Notably, co-culturing NT-T with DAC-induced U937-A2^+^ cells showed minor activation (Fig. 2A, E, I). We speculated that AML cell receiving DAC treatment might stimulate other antigen upregulations, including NKG2D ligands (NKG2DL) [38–40]. A subset of the T cells in bulk NT-T contained cytotoxicity T cells (CD8^+^, CD4^+^) and NK cell receptors (CD56^+^, NKG2D^+^) (Supplementary Fig. S4A–C). Therefore, the increased lysis of U937-A2^+^ cells by NT-T with DAC treatment may be associated with the upregulation of NKG2DL (Supplementary Fig. S4D-F), thereby facilitating the recognition and killing by T cells with NKG2D receptor.

### The enhanced anti-leukemia activity of NY-ESO-1-specific TCR-T is MHC-dependent

To determine whether the anti-leukemia activity of NY-ESO-1-specific TCR-T cells was MHC dependent, we incubated DAC-treated U937-A2^+^, HL60-A2^+^, and Kasumi-1-A2^+^ cells with an MHC class I mAb (W6/32 mAb) or isotype control (IgG2a). A high level of TNF-α secretion was observed in TCR-T cells incubated with DAC-treated target cells without the MHC class I mAb compared to NT-T cells (Supplementary Fig. S5A–C). After blocking the interaction between the peptide/HLA complex and TCR on the target cells by the MHC class-I antibody, the co-culture of NY-ESO-1-specific TCR-T cells with DAC-treated target cells showed a significant reduction in TNF-α secretion in response to the target cells. No significant change was observed in the secretion of TNF-α in the culture with untreated target cells (Supplementary Fig. S5D–F). Thus, the increased level of TNF-α production of TCR-T cells induced by DAC depends on the TCR recognition of peptide–MHC.

### NY-ESO-1-specific TCR-T cells recognize and kill primary leukemia blasts derived from DAC-treated AML patients

**Fig. 3:**
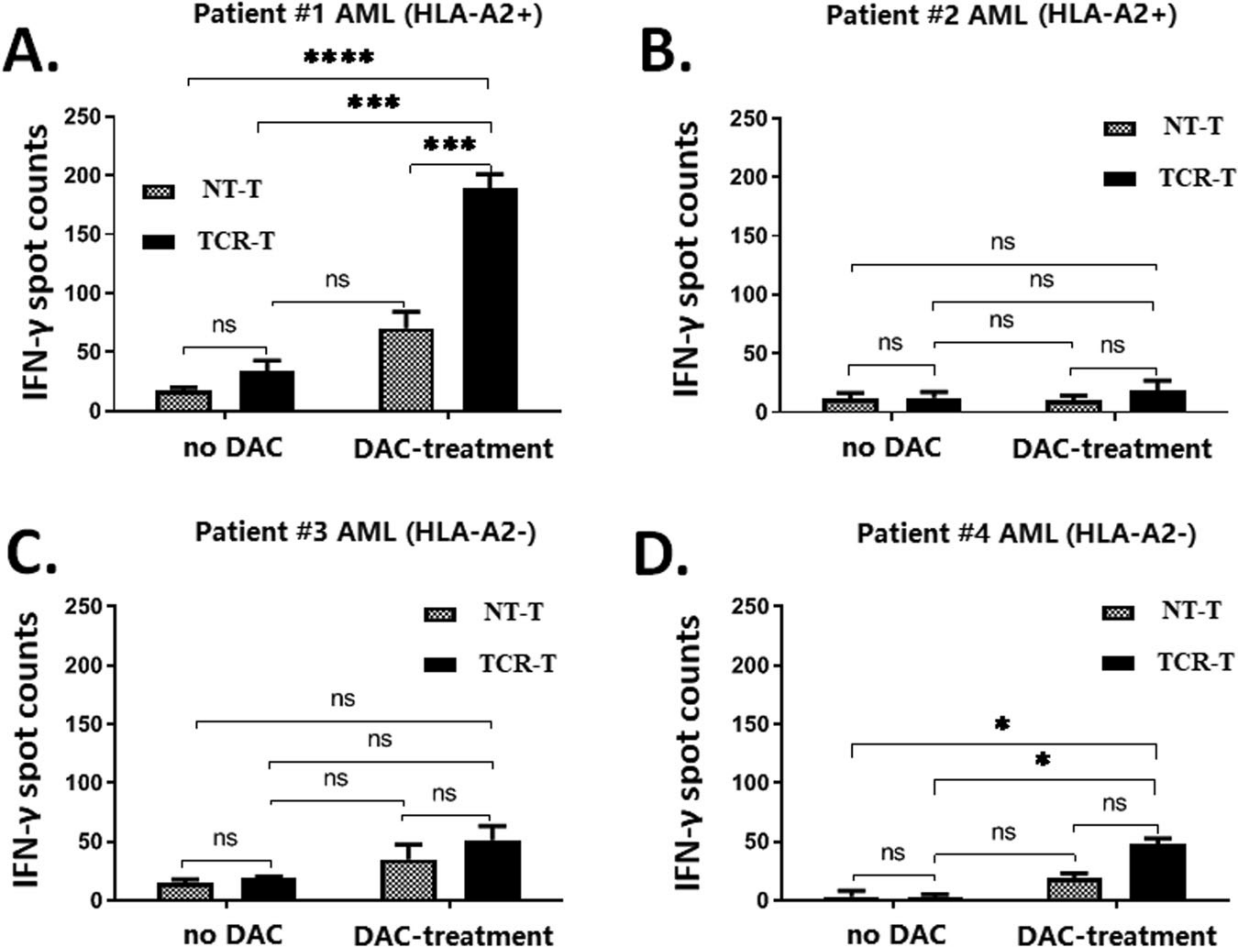
NY-ESO-1-specific donor-derived T cells effectively kill decitabine-treated primary AML blasts in vitro. IFN-γ ELISpot analysis was used to determine the magnitude of the NY-ESO-1-specific donor-derived T cell response to AML blasts from four patients. Briefly, 2 × 10^4^ AML blats from (**A**) patient #1 (HLA-A2^+^), (**B**) patient #2 (HLA-A2^+^; complete remission), (**C**) patient #3 (HLA-A2^-^), and (**D**) patient #4 (HLA-A2^-^) were co-cultured with either NT-T or TCR-T cells at an effector-to-target (E:T) ratio of 1:10 for 20 h. The data are presented as mean ± sd (*n* = 3). Statistical comparisons between two groups were determined by two-tailed unpaired *t* tests. *P < 0.05; ***P < 0.001; ****P < 0.0001; *ns* not significant.

We further accessed the cytolytic function of NY-ESO-1-specific donor-derived TCR-T cells against primary AML blasts derived from the bone marrow of four AML patients (Table 1). A NY-ESO-1-specific donor-derived TCR-T cell response was observed in the primary blasts of patient no. 1 with the HLA-A2^+^ genotype (Fig. 3A). And the co-culture of NY-ESO-1-specific donor-derived TCR-T cells with primary blasts treated with DAC produced a significantly higher level of IFN-γ than those stimulated by primary blasts without DAC treatment. A low TCR-T response was detected in the bone marrow of patient no. 2 with the HLA-A2^+^ genotype who achieved complete remission (Fig. 3B). Moreover, there was only a weak (non-specific) response in patients no. 3 and no. 4 who were carrying the HLA-A2^-^ genotype (Fig. 3C, D). Thus, the response of NY-ESO-1-specific TCR-T cells to DAC-treated AML cells is restricted by HLA.

### Decitabine exposure during TCR transduction further enhances the anti-leukemia efficacy of NY-ESO-1-specific TCR-T cells and promotes the development of the memory phenotype

**Fig. 4:**
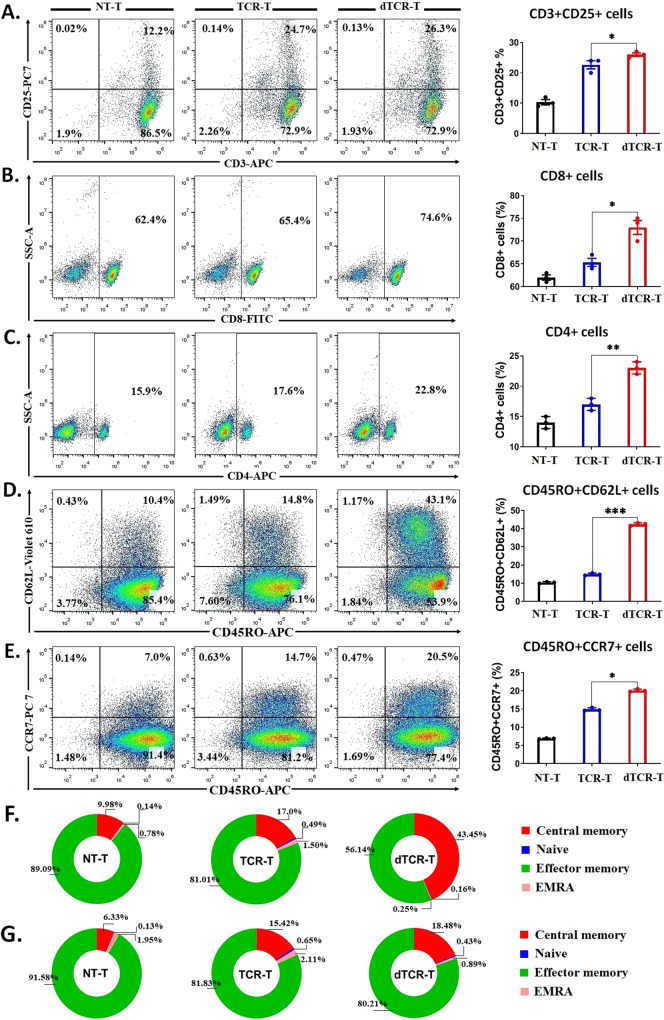
dTCR-T cells exhibit an improved memory phenotype under NY-ESO-1-specific stimulation. Phenotypic representation of (**A**) activation-associated CD3^+^CD25^+^ T cells, cytotoxic (**B**) CD8^+^ and (**C**) CD4^+^ cells, and central memory (**D**) CD45RO^+^CD62L^+^ T cells, and (**E**) CD45RO^+^CCR7^+^ T cells of NT-T, TCR-T, and dTCR-T cells from 12 days of the bulk cell culture (three healthy donors). **F**, **G** Pie chart showing the proportion of T-cell subsets, including central memory (CM; CD45RO^+^CD62L^+^/CCR7^+^), naive (CD45RO^-^CD62L^+^/CCR7^+^), effector memory (EM; CD45RO^+^CD62L^-^/CCR7^-^), and highly differentiated effector memory (EMRA; CD45RO^-^CD62L^-^/CCR7^-^) cells from panels D and E. The data are presented as mean ± sd (*n* = 3). Statistical comparisons between dTCR-T and TCR-T groups were determined by two-tailed unpaired *t* tests. *P < 0.05; **P < 0.01; ***P < 0.001.

Recently, it was demonstrated CAR-T cells treated with low-dose DAC exhibit improved expansion, enhanced cytotoxicity and cytokine production, as well as reduced exhaustion after antigen exposure [41] Based on these findings, we investigated whether DAC exposure during TCR transduction could enhance the proliferation, viability, longevity, and anti-leukemia activity of TCR-T cells. To produce long-term expression of TCR-T and increase memory-associated T cells, we treated the activated-T cells with low-dose DAC and lentiviral particle encoding TCR gene in the context of recognizing NY-ESO-1_157-165_ peptide. Lentivirus transduction, an efficient method for delivering transgenes to mammalian cells, achieved high transduction efficiency in the genome of various cell types, including CTLs and PBLs [42]. On the day three of TCR transduction, the culture was treated with DAC ranging from 10 to 1000 nM (Supplementary Fig. S6A), and these cells were designated as dTCR-T cells. A low-dose DAC (10–50 nM) showed little or no effect on the proliferation (Supplementary Fig. S6B) and viability of dTCR-T cells (Supplementary Fig. S6C), and showed no effect on generating the TCR-positive population (Supplementary Fig. S6D, E). By contrast, a high-dose DAC (200 nM or higher) showed toxicity with a significant reduction in the proliferation and viability of dTCR-T cells. The DNMT3a protein level was significantly reduced in dTCR-T cells compared with TCR-T cells after DAC treatment (Supplementary Fig. S6F), confirming the demethylation effect of DAC. Regarding the phenotypic changes, DAC (50 nM) increased the proportions of activated T cells (CD3^+^CD25^+^; Fig. 4A), cytotoxic phenotypes (CD8^+^ T cells and CD4^+^ T cells; Fig. 4B, C), and central memory-like phenotypes (CD45RO^+^CD62L^+^ and CD45RO^+^CCR7^+^ cells; Fig. 4D–G). Moreover, dTCR-T cells secreted higher levels of IFN-γ (Supplementary Fig. S6G) and TNF-α (Supplementary Fig. S6H) compared with TCR-T cells in the absence of target cells, indicating an enhanced unspecific killing capacity.

**Fig. 5:**
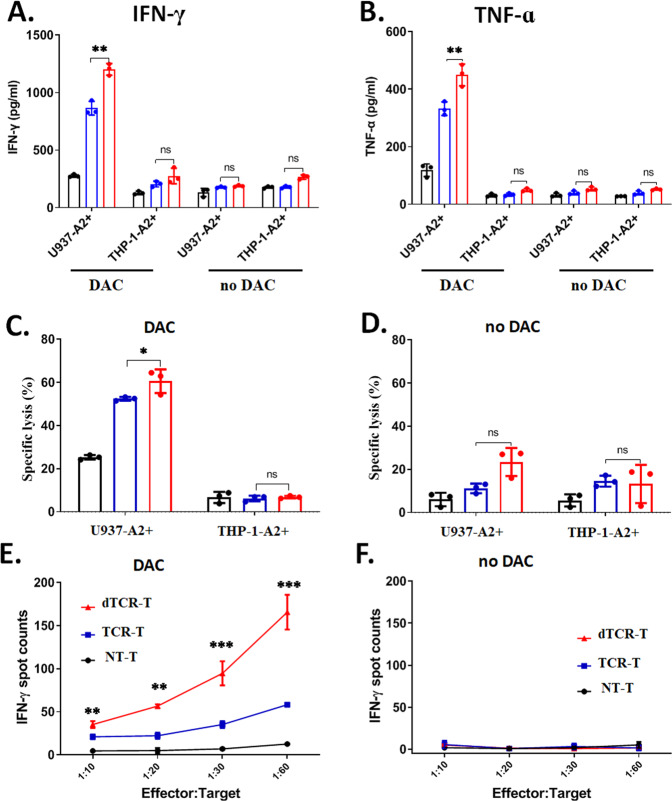
dTCR-T cells exhibit enhanced anti-leukemia cytotoxicity under NY-ESO-1-specific stimulation. ELISA assays analysis of (**A**) IFN-γ and (**B**) TNF-α secretion by the bulk cell culture of NT-T, TCR-T, and dTCR-T cells (from three healthy donors) that were stimulated by 2 × 10^4^ target cells (U937-A2^+^ with DAC-treated or untreated, and THP-1-A2^+^ with DAC-treated or untreated) at an effector-to-target (E:T) ratio of 5:1 for 20 h. **C**, **D** Increased cytotoxicity of dTCR-T against DAC-treated or untreated AML target cells. A total of 1 × 10^5^ effector cells of the bulk cell culture of NT-T, TCR-T, and dTCR-T were stimulated with DAC-treated or untreated target cells at an E:T ratio of 5:1 for 20 h. **E**, **F** Enhanced cytokine secretion of dTCR-T against DAC-treated or untreated 2 × 10^4^ U937-A2^+^ cells at serial E:T ratios (1:10, 1:20, 1:30, and 1:60) for 20 h. The data are presented as mean ± sd (*n* = 3). Statistical comparisons between dTCR-T and TCR-T groups were determined by two-tailed unpaired t tests. *P < 0.05; **P < 0.01; ***P < 0.001.

We assessed the in vitro functional activities of TCR-T cells and dTCR-T cells in the presence of DAC (50 nM) by ELISA and ELISpot assays. The productions of IFN-γ and TNF-α were further increased in dTCR-T cells cultured with DAC-treated AML cells (except for THP-1-A2^+^) (Fig. 5A, B; Supplementary Fig. S7). Consistent with the results of the cytokine release assays, dTCR-T cells exhibited enhanced in vitro cytotoxicity against DAC-treated U937-A2^+^ cells, but not THP-1-A2^+^ cells (Fig. 5C, D). Notably, dTCR-T cells demonstrated a high level of IFN-γ secretion against DAC-treated U937-A2^+^ cells, even at low E:T ratios (1:10, 1:20, 1:30, 1: 60) compared with TCR-T cells with equivalent TCR affinity (Fig. 5E). No response was observed in the untreated the target cells (Fig. 5F). These results indicate that DAC treatment during TCR signaling transduction can enhance the anti-leukemia activity of NY-ESO-1-specific dTCR-T cells against AML and promote the development of the memory-like phenotypes.

### NY-ESO-1-specific dTCR-T cells prolong the survival of AML xenograft mouse model with enhanced effector function and memory phenotype production

**Fig. 6:**
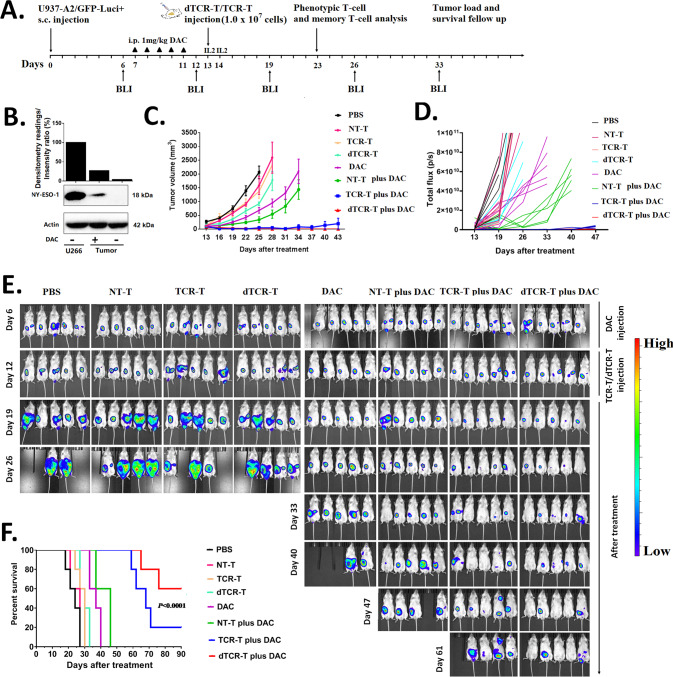
NY-ESO-1-specific dTCR-T cells exhibit enhanced in vivo anti-tumor activity in the decitabine-treated AML xenograft mouse model. **A** In vivo experimental layout. A total of 3 × 10^6^ fluorescence-expressing U937-A2-luciferase^+^ cells in PBS were s.c. transferred to NCG mice. Tumor growth was monitored by BLI with the depicted regimen. After engraftment was confirmed on day 7, the animals were organized into eight groups according to tumor size (average ~80–100 mm^3^), including four groups that were treated with PBS (*n* = 5), NT-T cells (*n* = 5), TCR-T (*n* = 5), and dTCR-T cells (*n* = 5), and another four groups that were treated with DAC alone (*n* = 5) or in combination with adoptive T cells (NT-T plus DAC, *n* = 5); TCR-T plus DAC, *n* = 5); and dTCR-T plus DAC, *n* = 5). DAC was given on days 7–11 via i.p. injection at a dose of 1.0 mg/kg body weight in 100 μL of PBS. On day 13, mice (except for PBS and DAC groups) were treated by i.v. injection of 1.0 × 10^7^ NT-T cells, TCR-T cells, or dTCR-T cells. Tumor volume was assessed every 3 days using calipers with the formula (length × width^2^)/2) and weekly by BLI with the IVIS Lumina III System. **B** Western blot analysis of the expression of NY-ESO-1 protein in tumor tissues 3 days after the last administration of DAC, with the multiple myeloma cell line U266 as the positive control (*n* = 3). **C** Mean tumor growth curves of the eight groups (*n* = 5). **D** Qualification of the BLI signal from each treatment group over the time (*n* = 5). Data are represented as the total fux (photons/second). **E** BLI images showing tumor burdens in all mice at the indicated time points (*n* = 5). **F** Kaplan–Meier survival curves presenting the overall survival of each group (*n* = 5). The log-rank Mantel–Cox test was used to analyze the survival of each group.

To investigate the efficacy of NY-ESO-1-specific TCR-T cells against DAC-treated AML cells in vivo, we established an AML xenograft mouse model using GFP-encoding U937-A2-luciferase^+^ cells (Fig. 6A). The expression of NY-ESO-1 protein was detected in the tumor tissues derived from DAC-treated mice (3 days after DAC treatment), but it was undetectable in untreated tissues (Fig. 6B). TCR-positive cells comprised ~57% of the bulk cell culture of TCR-T cells and dTCR-T cells (Supplementary Fig. S6D). Tumor growth was uncontrolled in mice that received PBS, NT-T, TCR-T, or dTCR-T cells without DAC (Fig. 6C–E). The administration of DAC alone slowed the tumor growth rate. Among the combined treatment regimens, mice treated with NT-T cells plus DAC showed further reduced tumor growth compared with DAC alone. The combination of DAC with TCR-T cells significantly suppressed tumor development, but tumor recurrence appeared around 1 month after treatment (43 days in Fig. 6C–E). The combination of dTCR-T plus DAC showed a superior tumor suppressive effect compared with the other treatment regimens. On day 61, three out of five mice treated with NY-ESO-1-specific dTCR-T cells plus DAC were tumor-free, as revealed by IVIS, while most mice in the TCR-T plus DAC group exhibited signs of tumor relapse or recurrence (Fig. 6E). Moreover, the survival of mice in the dTCR-T plus DAC group was significantly prolonged compared with the TCR-T plus DAC group and the other control groups (Fig. 6F). Interestingly, three out of five mice in the dTCR-T plus DAC group showed complete response until the end of the study (over 90 days).

**Fig. 7:**
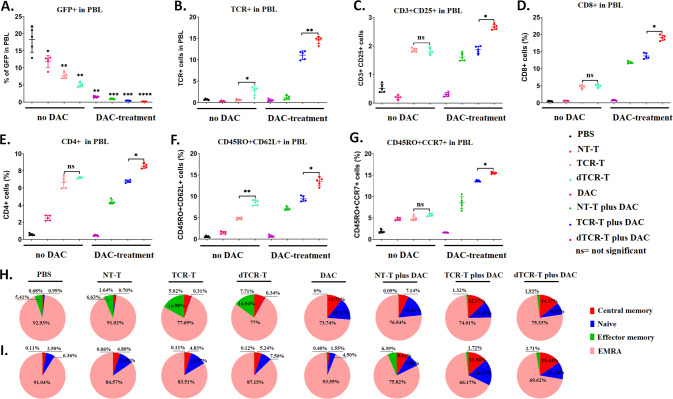
The combination of dTCR-T cells with decitabine produces more cytotoxic effector cells and cells with the memory-like phenotype in vivo. After 10 days of treatment (on day 23), the peripheral blood of five mice from each group was analyzed for (**A**) GFP^+^ cells, (**B**) TCR^+^ cells, (**C**) activated CD3^+^CD25^+^ T cells, cytotoxic (**D**) CD8^+^ and (**E**) CD4^+^ T cells, and central memory (**F**) CD45RO^+^CD62L^+^ and (**G**) CD45RO^+^CCR7^+^ T cells by flow cytometry. **H**, **I** Pie chart showing the proportion of T-cell subsets, including central memory (CM; CD45RO^+^CD62L^+^/CCR7^+^), naive (CD45RO^-^CD62L^+^/CCR7^+^), effector memory (EM; CD45RO^+^CD62L^-^/CCR7^-^), and highly differentiated effector memory (EMRA; CD45RO^-^CD62L^-^/CCR7^-^) cells from panels F and G. The data are presented as mean ± sd (*n* = 5). Statistical comparisons between two groups were determined by two-tailed unpaired t tests. *P < 0.05; **P < 0.01; ***P < 0.001; *ns* not significant.

To interpret the enhanced anti-tumor activity of the combined treatment of dTCR-T plus DAC in vivo, flow cytometry was employed to analyze the percentages of GFP-positive, TCR-positive cells, and T-cell phenotypes in the peripheral blood of mice 10 days after treatment with DAC. The number of GFP^+^ cells in peripheral blood was significantly reduced in the three groups that received T cells plus DAC (Fig. 7A), which corresponded to the tumor size of each group. A higher percentage of TCR-positive cells in peripheral blood were observed in mice treated with TCR-T plus DAC, which was further increased in those treated with dTCR-T cells plus DAC (Fig. 7B). And mice treated with dTCR-T cells plus DAC showed a higher proportion of activated CD3^+^CD25^+^ cells (Fig. 7C), CD8^+^ cells (Fig. 7D), and CD4^+^ cells (Fig. 7E) compared with the other groups, suggesting higher anti-leukemia activity.

In addition, we measured the percentages of central memory T cells in the peripheral blood of the treated animals. Mice treated with dTCR-T cells showed 8.75% of CD45RO^+^CD62L^+^ T cells, which was significantly higher than that of the other groups (4.82%, TCR-T; 1.52%, NT-T; 0.57%, PBS; Fig. 7F, H), while the percentages of CD45RO^+^CCR7^+^ T cells showed no significant differences among dTCR-T, TCR-T, and NT-T treatment groups (Fig. 7G, I). The percentages of both types of central memory T cells were significantly increased in mice that received DAC with dTCR-T, TCR-T, or NT-T cells. Among the four DAC-treated groups, the highest percentages of CD45RO^+^CD62L^+^ (13.48%) and CD45RO^+^CCR7^+^ T cells (15.49%) were found in mice treated with dTCR-T plus DAC.

Taken together, a combination of DAC with NY-ESO-1-specific dTCR-T cells is superior in suppressing AML xenograft tumor growth and promoting tumor-free survival, possibly by promoting the maintenance of effector function and the memory phenotype.

## Discussion

DAC exerts anti-tumoral activity via several potential mechanisms that associate with demethylation, including cytotoxicity and the DNA damage response [43, 44], re-expression of aberrantly silenced tumor suppressor genes [45, 46], upregulation of silenced tumor-associated antigens that enhance the anti-tumor immune response [47], and induction of cytosolic sensing of the double-stranded RNA response [48]. Among the up-regulated genes induced by DAC in AML blasts, NY-ESO-1 attracted our attention by its restricted tissue expression and immunogenicity suggesting a potential immunotherapeutic target to treat AML. In fact, NY-ESO-1-specific TCR-T cells have demonstrated high efficacy against both hematologic malignancies and solid tumors [16, 18, 19, 21]. Therefore, we anticipated that the combination of NY-ESO-1-specific TCR-T cells with DAC might achieve significant anti-leukemia potential in the treatment of AML.

Myeloid leukemia shows an absence or very low level of NY-ESO-1 expression due to dense promoter hypermethylation [22]. Consistent with previous studies [25, 49, 50], our results demonstrated that DAC could induce NY-ESO-1 re-expression in human AML cell lines through DNA promoter demethylation. Nevertheless, the DAC-induced expression of NY-ESO-1 protein was not detected in the THP-1 cell line as reported previously [23]. We postulate that the non-response was caused by the immature morphology of CpG methylation patterns of the THP-1 cell line [51], as it was established from the peripheral blood of a 1-year-old boy with AML. In addition to these results, the primary AML blasts derived from patients who received DAC treatment also showed increased NY-ESO-1 expression, consistent with a previous study [25]. Moreover, the bone marrow of an AML patient with a complete response expressed a low level of NY-ESO-1 after DAC treatment, confirming that DAC-induced NY-ESO-1 expression is restricted to AML blasts. Thus, the quantification of NY-ESO-1 expression in patients who received DAC may predict the clinical benefits of this combination approach.

The present study showed that the induced NY-ESO-1-expressing in AML cells could be efficiently targeted and killed by NY-ESO-1-specific TCR-T cells, both in vitro and in AML xenograft mouse models. The TCR-transduced T cells specifically recognized NY-ESO-1 in the context of the HLA-A2 restricted NY-ESO-1_157-165_ peptide, consistent with a previous report [23]. The inhibition of target cells with an MHC-class I monoclonal antibody showed attenuated TCR activity with a significant reduction of TNF-α production by the NY-ESO-1-specific TCR-T cells. Our previous study demonstrated the effect of soluble high-affinity TCR (26 pM) in blocking melanoma and multiple myeloma cell lines, which could significantly reduce IFN-γ secretion [32]. Therefore, these data confirm that the enhanced anti-leukemia capacity of NY-ESO-1-specific TCR-T cells is MHC-dependent.

The combination of DAC with high-affinity NY-ESO-1-specific TCR-T cells revealed significant anti-leukemia efficacy in vivo, with complete tumor eradication and prolonged survival of AML xenograft mouse models. However, tumor relapse was still inevitable, indicating that the TCR-T cells might not persist long enough to maintain long-term remission. It was recently reported that DAC could induce DNA reprogramming of CAR-T cells, thereby promoting sustained cell expansion, cytotoxicity, and cytokine production, while reducing exhaustion after antigen exposure [41]. Based on these findings, we wondered whether TCR-T cells exposed to DAC during transduction could further increase their anti-leukemia capacity, proliferation, and longevity, and promote tumor-free survival. Central memory T cells, which are circulating cells prevalent in lymph nodes, have enhanced longevity and proliferative potential. As expected, treating TCR-T cells with a low dose of DAC (50 nM) during transduction, which were designated as dTCR-T cells, further enhanced their anti-leukemia capacity and cytokine secretion, and promoted the memory phenotype, which corresponded to an increased proportion of cytotoxic CD4^+^/CD8^+^ T cells and central memory CD45RO^+^CD62L^+^/CD45RO^+^CCR7^+^ T cells, respectively. Compared with TCR-T cells, the dTCR-T cells further prolonged the survival of DAC-treated mice bearing AML xenograft tumors. Consistent with previous studies [41, 52, 53], the development of the memory phenotype can boost anti-tumor immunity and ACT persistence, thereby resulting in superior clinical outcomes. Taken together, the increased anti-leukemia activity of dTCR-T cells could be related to an up-regulation in the cytotoxicity of T cells and an increase in the proportion of memory phenotype T-cells owing to the DAC-induced DNA methylation.

A safety concern of this combination is the potential risk of up-regulated CTA expression in normal human tissues induced by demethylation drugs. Several studies have demonstrated that DAC has no side effects on presenting antigen targets, including NY-ESO-1 in normal human tissues and cells such as normal skin, colon, bronchial epithelia, bone marrow, peripheral blood, astrocytes, fibroblasts, smooth muscle, and ovaries (Table 2) [54–59]. Moreover, our in vitro study demonstrated limited NY-ESO-1 expression in normal bone marrow cells and normal cell lines after treatment with DAC (Supplementary Fig. S2). The co-culture of these cells with high-affinity NY-ESO-1-specific TCR-T did not lead to cytotoxicity or cytokine secretion (Supplementary Fig. S8). Equally important, the therapeutic potential and safety of NY-ESO-1 vaccination in combination with DAC has been demonstrated in phase I clinical trials for the treatment of patients with high-risk MDS and ovarian cancer [27, 59]. These data are essential for prospective studies that aim to correlate the induction of NY-ESO-1-specific TCR-T or dTCR-T cells with the clinical response in patients with hematopoietic malignancies such as AML treated with DAC. However, additional clinical trials are necessary to access the safety of DAC combined with high-affinity TCR-T.

**Table 1:** Characteristics of the enrolled AML patients.

Patient ID	Age	Sex	Diagnosis	Karyotype	Clinical treatment (mg/m^2^/day)	HLA subtype	Blast counts (%)	
							Diagnosis	Post-treatment
Patient #1	29	M	AML-M2	Normal	DAC 20 mg/d d1-5 × 4 courses	HLA-A2^+^	94.5%	73.7%
Patient #2	31	F	AML-M2	Normal	DAC 20 mg/d d1-5 × 4 courses	HLA-A2^+^	62.5%	4.5%
Patient #3	68	F	AML-M2	Normal	DCAG	HLA-A2^-^	64%	39.2%
Patient #4	60	F	AML-M5	Normal	DCAG + Chidamide 20 mg/d d1-5 × 4	HLA-A2^-^	73%	16.3%

*d* day, *DAC* decitabine, *DCAG* DAC 20 mg/d + aclarubicin 20 mg/d, cytarabine 100 mg/12 h d1-5 × 4 courses

The predominant mechanism of action of DAC is likely dose-dependent, with low doses inducing silenced gene re-expression and minimal DNA damage, while high doses causing more pronounced DNA damage and apoptosis. In clinical settings, low-dose DAC is recommended for the treatment of MDS and AML to favor hypomethylation over cytotoxicity. It has been reported that a low dose (0.25 mg/kg) of a demethylating agent could exert robust anti-tumor effects on hematological and epithelial tumor cells, and a significant reduction in tumor size was observed in mice bearing established xenografts after three cycles (2 weeks/cycle) of treatment [60]. Data from an in vitro study suggested that the DAC-induced expression of NY-ESO-1 protein was presented in time- and dose-dependent manners [23]. Thus, the DAC dosage and treatment regimen deserve further investigation to maximize and sustain the expression of NY-ESO-1 protein, thereby optimizing the TCR-T response.

In summary, DAC-induced NY-ESO-1 can be used as an immune target for TCR-T to treat AML. High-affinity NY-ESO-1_157-165_ TCR-T cells showed an efficient anti-AML response. Demethylating TCR-T cells with DAC during transduction (dTCR-T) can significantly increase their cytotoxicity and cytokine secretion after antigen exposure. dTCR-T cells outperformed TCR-T cells in the anti-leukemia effects and preventing recurrence, likely by producing a higher proportion of memory-like phenotypes. There are several limitations in the present study. (1) The safety of combined high-affinity TCR-T cells with DAC should be further evaluated on cells from more critical organs such as brain, heart, and liver. (2) The AML xenograft mouse model cannot recapitulate the molecular genetics and phenotypic features, as found in primary human AML. (3) Similar to most clinical studies, we generated the engineered T cell products from PBMCs, which were mixed T cell populations containing both highly functional T cells and less-differentiated phenotypes. In preclinical models, the use of purified, naive T cell subsets for adoptive immunotherapy can enhance persistence and anti-tumor immunity [61]. Thus, the anti-leukemia effects of NY-ESO-1-specific TCR-T cells may be further improved if starting from naïve T cells selected from PBMCs.

## Materials and methods

### Cell lines

The antigen NY-ESO-1_157–165_ (peptide SLLMWITQV) and HLA-A*02:01 double-negative cells of human acute myeloid leukemia (AML) cell lines (U937, HL60, and Kasumi-1), human normal cell lines (kidney proximal tubular 2, HK-2; breast epithelial cell, MCF10A), and the antigen NY-ESO-1_157–165_-negative and HLA-A*02:01 positive cells (THP-1) were purchased from ATCC (Manassas, VA, USA). Human normal colon epithelia cell line NCM460, the antigen NY-ESO-1_157–165_ and HLA-A*02:01 double-negative cells, was purchased from IN CELL (San Antonio, TX, USA). The antigen NY-ESO-1_157–165_ and HLA-A*02:01 double-positive cells of the multiple myeloma (MM) cell line U266 (used as a positive control for NY-ESO-1 expression) were purchased from CBTCCCAS (Shanghai, China). We obtained HLA-A*02:01-positive cells of U937, HL60, Kasumi-1, HK-2, NCM460, and MCF10A cell lines by transducing the cell lines with HLA-A*02:01 lentiviral particles. AML and MM cell lines were cultured in RPMI 1640 (Gibco Life Technologies, Grand Island, NY, USA) containing 10% fetal bovine serum (FBS; Gibco Life Technologies). HK-2 cells were cultured in Minimum Essential Medium (MEM, Gibco Life Technologies, Grand Island, NY, USA) containing 10% FBS. MCF10A cells were cultured in DMEM-F12 (Procell, Wuhan, China) supplemented with 5% horse serum, 20 mg/mL epidermal growth factor (EGF), 0.01 mg/mL insulin, and 0.5 μg/mL Hydrocortisone. 293 T cells, a human embryonic kidney cell line purchased from ATCC. 293 T and NCM460 cells were cultured in Dulbecco’s Modified Eagle Medium (DMEM, Gibco Life Technologies) containing 10% FBS.

### Patient samples

The use of human materials in this study was approved by the Institutional Review Board of the Shenzhen University General Hospital. Mononuclear cells from the bone marrow of AML patients or healthy donors were isolated and cryopreserved following Ficoll centrifugation (Ficoll-Paque, GE Healthcare, Björkgatan, Uppsala, Sweden). The characteristics of the enrolled patients in clinics are shown in Table 1.

**Table 2:** Restricted NY-ESO-1 gene expression in normal cells or tissues treated with the decitabine.

Type of therapy	Type of cancer	Type of normal cells or tissues and clinical study	Side effects	Reference
High-affinity NY-ESO-1_157-165_ TCR-T cells plus decitabine	Acute myeloid leukemia	AML blasts, normal cell lines; in vitro	Undetectable NY-ESO-1mRNA and protein levels	Present study
NY-ESO-1-specific T cells plus decitabine	Multiple myeloma	Clinical study (NCT01050790)	Safe and feasible	[54]
NY-ESO-1_157-165_ TCR-T cells plus decitabine	Colorectal cancer	Fibroblasts (skin, colon)	Negligible effects	[55]
NY-ESO-1_157-165_ TCR-T cells plus decitabine	Thoracic cancer	Bronchial epithelia	Safe and feasible	[56]
NY-ESO-1specific CTLs plus decitabine	Glioma	Astrocyte cells,	Undetectable NY-ESO-1 in human normal cells	[58]
NY-ESO-1 specific CD8 + T cells, CD4 + T cells; NY-ESO-1 Vaccine plus decitabine	Ovarian cancer	Clinical study (NCT00887796)	Safe and feasible	[59]
DEC-205/NY-ESO-1 specific CD8 + T cells, CD4 + cells NY-ESO-1 Vaccine plus decitabine	Myelodysplastic syndrome (MDS) or acute myeloid leukemia (AML)	Clinical study (NCT 01834248)	Safe and feasible	[62]

### Decitabine treatment

5-Aza-2’-deoxycytidine (DAC; Sigma-Aldrich) was dissolved in phosphate buffered saline (PBS, 100 µM stock) and stored at −80 °C. We tested different doses and times of DAC treatment to optimize NY-ESO-1 expression in AML cells. AML cell lines were treated with 100 nM, 200 nM, 500 nM, or 1000 nM DAC in the cell culture medium for 72 h. For the time-dependent study, AML cell lines, normal cell lines (HK-2, NCM460, and MCF10A), and normal bone marrow cells were treated with 200 nM DAC, and NY-ESO-1 expression was measured on days 2, 3, 5, 7, and 10.

### RNA extraction, cDNA synthesis, and real-time PCR

Total RNA was isolated with TRIzol Reagent (Invitrogen, Carlsbad, CA, USA) and synthesized from 1 μg of total RNA with the TransScript All-in-One First-Strand cDNA Synthesis SuperMix Kit for qPCR (Transgen, Beijing, China) according to the manufacturer’s protocol. Quantitative RT-PCR was performed with the 7500 Real-Time PCR System (Thermo Fisher Scientific, Waltham, MA, USA) using the *PerfectStart* SYBR Green qPCR SuperMix Kit (Transgen). The primers for NY-ESO-1 and β-actin, which were used in this study, have been described previously [33]. The NY-ESO-1 primer sequences were as follows: forward primer – 5’-AAAAACACGGGCAGAAAGC-3’ and reverse primer – 5’-GCTTCAGGGCTGAATGGAT-3’. The β-actin primer sequences were as follows: forward primer – 5’-CCTCCATGATGCTGCTTACATGTC-3’ and reverse primer – 5’-ATGTCTCGCTCCGTGGCCTTAGCT-3’. The TCR primer sequences were as follows: forward primer – 5’-ATGGAGACACTGCTGGGC-3’ and reverse primer – 5’-CATGGTGAAGAAGAAGAACAGCTAA-3’. The relative expression of NY-ESO-1 and TCR was normalized to β-actin and calculated using the 2^-ΔΔCt^ method [34].

### Western blotting

Cells and tumor tissues were lysed in RIPA buffer containing protease inhibitors (Solarbio, Beijing, China). Proteins were size-fractionated on 10–12% PAGE gels and transferred to Immobilon-transfer nitrocellulose membranes (Millipore, Burlington, MA, USA). The membranes were incubated with anti-human NY-ESO-1 (1:250 dilution; Clone SP349; Abcam, Cambridge, MA, USA), anti-DNMT3 (1:2000; Clone EPR18455, Abcam), and anti-β-actin (1:2000 dilution; Clone 13E5; Cell Signaling Technology, Danvers, MA, USA), washed, and incubated with an HRP-labeled secondary antibody. Protein bands were detected with the Chemiluminescent HRP Substrate Kit (Millipore). The densitometry readings/intensity ratios were analyzed with the ChemiDoc XRS + System (Bio-Rad, Hercules, CA, USA) by comparing the protein band intensity of NY-ESO-1 or DNMT3a to the protein band intensity of β-actin.

### DNA methylation analysis

Genomic DNA was isolated from DAC-treated AML cell lines and untreated control cells with the Wizard Genomic DNA Purification Kit (Cat. A1120, Promega, Madison, WI, USA). The sodium bisulfite conversion of DNA was performed with the EZ DNA Methylation Kit (Cat. D5001, Zymo Research, Irvine, CA, USA). The methylation status of the NY-ESO-1 promoter region was accessed by sodium bisulfite sequencing as described previously [35]. MethPrimer was used to design the primers [36], and the primer sequences were as follows: forward primer – 5’-GGATGGGATAGGTTGGGTTT-3’ and reverse primer – 5’-AACTTAAACCCCTCACCCCTA-3’. The PCR products were purified and cloned into the pGM-T vector (Cat.VT202-01, TianGen, Beijing, China). The individual clones were analyzed using the ABI PRISM 3730 DNA Analyzer (Applied Biosystems, Foster, CA, USA).

### Flow cytometry

Multicolor gated flow cytometry was performed to analyze the expression of cell surface proteins stained with monoclonal antibodies (mAbs) as described previously [32]. The human mAbs used in this study are listed in Supplementary Table 1. CytoExpert software (version 1.1.10.0, Beckman Coulter, Brea, CA, USA) and FlowJo software (Version 10.0.7, FlowJo LLC, Ashland, OR, USA) were used to analyze the FACS data.

### Generation of TCR-T cells

NY-ESO-1-specific high-affinity 1G4 TCR (K_D_ = 1.07 μM) and wild-type 1G4TCR (K_D_ = 32 μM) in the context of HLA-A*02:01 was generated and cloned into a lentivirus vector as described previously [32]. The unique clone of wild-type 1G4 TCR, which was isolated from a melanoma patient, was substituted with a dual amino acid in the third complementary determining region (CDR3α) to enhance the TCR affinity and improve the antigen-specific reactivity of T cells (designated 1G4-α95:LY TCR) [37]. Lentiviral particle production and generation of NY-ESO-1-specific TCR-T cells were performed as described previously [32]. To produce the lentiviral products, the desired TCR genes (containing α- and β-chains) were cloned into the pCDH lentiviral vector. The desired TCR plasmid and lentiviral package system consisting of the packaging construct (RRE), Rev expression plasmid (REV), and envelope construct (pG2M.D) were transfected into ~80% confluent 293 T cells, which were cultured in DMEM containing 10% FBS. The supernatant was collected after 48 and 72 h of transfection, concentrated by ultracentrifugation (50 kDa centrifugal filter units; Merck kGaA, Darmstadt, Germany) at 4000 g for 20 min at 4 °C, and stored at −80 °C.

For the generation of NY-ESO-1 specific TCR-T cells (TCR-T), human peripheral blood cells (PBMCs) from healthy donors were stimulated with human T-activator CD3 dynabeads/CD28 mAb microbeads (Cat.11161D, Life Technologies, Grand Island, NY, USA) at a bead:cell ratio of 1:1, and cultured in X-VIVO 15 medium (Lonza, Basel, Switzerland) containing 100 IU/mL IL-2 (PeproTech Inc., Beijing). After 48 h of activation, activated T cells (1 × 10^6^) were transduced with a multiplicity of infection of 5 (5 MOI) of lentiviral particles containing the TCR gene at 48 and 72 h.

To examine the effects of DAC on the development of TCR-T, after 48 h of PBMC stimulation, serial doses (10 nM, 50 nM, 200 nM, and 1000 nM) of DAC were added simultaneously during the first 24 h of transduction. T cells were then re-transfected with TCR lentiviral particles without DAC for another 24 h (designated as dTCR-T cells). After transduction, the bulk TCR-T, dTCR-T, and NT-T cells were expanded ex vivo with X-VIVO 15 medium containing IL-2.

The dynabeads were removed before cell analysis. TCR-positive T cells were detected by staining with FITC-, PE-, and APC-conjugated antibodies, including anti-human CD8 (Clone RPA-T8, BioLegend, San Diego, CA, USA), anti-human CD3 (Clone HIT3a, BioLegend), and anti-human TCR vβ13.1 (Clone H131, BioLegend) or anti-mouse TCR β chain (Clone H57-597, BioLegend).

### Enzyme-linked immunosorbent assay (ELISA)

Cytokine production was assessed by ELISA (BioGems, Westlake Village, CA, USA) using supernatants according to the manufacturer’s protocol. Briefly, effector cells were cultured with 2 × 10^4^ target cells at an E:T ratio of 5:1 for 20 h in fresh medium (final volume, 200 μL). Supernatants and standard controls (100 μL, prepared according to the instructions) were transferred to a primary antibody pre-coated (IFN-γ or TNF-α) 96-well strip microplate, incubated at 37 °C for 90 min, and washed. The avidin-biotin-peroxidase complex was added, and the microplate was incubated at 37 °C for 30 min. After additional washes, the color-developing reagent was added, and the microplate was maintained in the dark for 30 min. The reaction was stopped, and the absorbance was measured with a Multiskan FC microplate reader (Thermo Fisher Scientific) at 450 nm.

### Enzyme-linked immunospot (ELISpot) assay

ELISpot assays were performed according to the manufacturer’s protocol (BD Biosciences, Franklin Lakes, NJ, USA; Bio-Techne, Minneapolis, MN, USA). Briefly, 96-well flat-bottom plates were pre-coated overnight with anti-IFN-γ, washed with RPMI 1640 containing 10% FBS, and blocked with culture medium at room temperature for 2 h. The effectors were added at a final concentration of 2 × 10^3^ cells per well in duplicate in the presence of target cells at serial effector-to-target (E:T) ratios (1:10, 1:20, 1:30, and 1:60) for 20 h. The biotinylated secondary antibody was added, and the plates were incubated at room temperature for 2 h. Streptavidin-horseradish peroxidase was added, followed by incubation for 1 h. After additional washes, 3-amino-9-ethylcarbazole (AEC Substrate Kit; BD Biosciences) was added, followed by incubation at room temperature for 3–5 min. The reaction was stopped by the addition of water, and the plate was air-dried and analyzed using the ELISpot reader (BIOsys ELISpot Reader, Karben, Germany).

### Lactate dehydrogenase (LDH) assay

Cytotoxicity was assessed with the CytoTox 96 Non-radioactive Cytotoxicity Assay (Promega) according to the manufacturer’s protocol. Briefly, effector cells were prepared in fresh medium and cultured with 2 × 10^4^ target cells at serial E:T ratios (5:1, 2.5:1, and 1.25:1) in a final volume of 200 μL. Control groups, such as spontaneous release (effector or target cells only), maximum release (target cells with 20 μL ×10 lysis buffer), and medium background (no cells added), were also set up. The plates were incubated at 37 °C for 20 h. After centrifugation, 50 μL of the supernatant was transferred into the wells of a flat-bottom plate, and 50 μL of CytoTox 96 Reagent was added. The plates were incubated at room temperature in the dark for 30 min. The reaction was stopped by adding 50 μL of stop solution, and the absorbance was measured with a Multiskan FC microplate reader at 490 nm. Cytotoxicity was calculated using the following formula:

$${{{\mathrm{\% }}}}\;{{{\mathrm{Cytotoxicity}}}} = \frac{{{{{\mathrm{Experimental}}}} - {{{\mathrm{Effector}}}}\;{{{\mathrm{Spontaneous}}}} - {{{\mathrm{Target}}}}\;{{{\mathrm{Spontaneous}}}}}}{{{{{\mathrm{Target}}}}\;{{{\mathrm{Maximum}}}}-{{{\mathrm{Target}}}}\;{{{\mathrm{Spontaneous}}}}}} \times 100$$

### Mouse xenograft models

Animal studies were performed using a standard protocol approved by the IACUC of Peking University Shenzhen Graduate School (Shenzhen, China). Immunodeficient NCG (NOD/ShiLtJGpt-Prkdcem^26Cd52^Il2rg^em26Cd22^/Gpt) mice (6 weeks old, male) were purchased from GemPharmatech Co., Ltd. (Nanjing, China). Each NCG mouse received a subcutaneous injection of 3.0 × 10^6^ fluorescent U937-A2-luciferase^+^ cells (U937-A2-GFP-Luci^+^) in 200 µL of PBS on day 0. Animals were organized into eight groups according to tumor size (average, 80–100 mm^3^), including four groups treated without DAC (PBS, *n* = 5; NT-T, *n* = 5; TCR-T, *n* = 5; and dTCR-T, *n* = 5), and another four groups treated with DAC alone (*n* = 5) or in combination with adoptive T cells (NT-T plus DAC, *n* = 5; TCR-T plus DAC, *n* = 5; and dTCR-T plus DAC, *n* = 5). DAC was given on days 7 to 11 via intraperitoneal (i.p.) injection at a dose of 1.0 mg/kg body weight in 100 μL of PBS. On day 13, mice in six groups (except PBS and DAC groups) were treated by intravenous (i.v.) injection of 1.0 × 10^7^ NT-T cells, TCR-T cells, or dTCR-T cells.

Tumor growth was monitored every 3 days using a caliper and weekly by bioluminescence imaging (BLI) with the IVIS Lumina III in Vivo Imaging System (PerkinElmer, Waltham, MA, USA). The prolonged survival of animals was recorded from the first day of treatment until death or the largest tumor volume (≤1800 mm^3^). Peripheral blood (~200 μL) was collected through the canthus for subsequent flow cytometry analysis of tumor cell infilration and TCR-T cell persistence.

### Statistical analyses

Statistical analyses were performed using GraphPad Prism 8.0 software (GraphPad Software, Inc., San Diego, CA, USA). Statistical analysis was performed using unpaired two-tailed Student’s *t*-test for comparing two groups and log-range Mantel–Cox test for comparing survival differences between the groups. *P* < 0.05 was considered statistically significant.

## Supplementary information

Supplementary materials
