# Supplementary material for: Decitabine enhances targeting of AML cells by NY-ESO-1-specific TCR-T cells and promotes the maintenance of effector function and the memory phenotype
Source: Oncogene. 2022 Sep 12;41(42):4696–708. doi: 10.1038/s41388-022-02455-y (PMC9568428; doi:10.1038/s41388-022-02455-y)
Supplement: Supplementary file 1 — Supplementary materials [file 41388_2022_2455_MOESM1_ESM.docx]

Decitabine enhances targeting of AML cells by NY-ESO-1-specific TCR-T cells and promotes the maintenance of effector function and the memory phenotype

Synat Kang^1^, Lixin Wang^1^, Lu Xu^1^, Ruiqi Wang^3^, Qingzheng Kang^1^, Xuefeng Gao^1,2,^*, Li Yu^1,^*

^1^Department of Hematology and Oncology, International Cancer Center, Shenzhen Key Laboratory of Precision Medicine for Hematological Malignancies, Shenzhen University General Hospital, Shenzhen University Clinical Medical Academy, Shenzhen University Health Science Center, Shenzhen 518000, Guangdong, China

^2^Central Laboratory, Shenzhen Key Laboratory of Precision Medicine for Hematological Malignancies, Shenzhen University General Hospital, Shenzhen 518000, Guangdong, China

^3^School of Medicine, Nankai University, Tianjin, China, 300071

**Correspondence to:* Xuefeng Gao, E-mail: xfgao@szu.edu.cn; Li Yu, E-mail: yuli@szu.edu.cn

**Supplementary Table S1.** Human conjugated monoclonal antibodies used in this study.

| Human conjugated monoclonal antibodies used in this study | | | | | |
| --- | --- | --- | --- | --- | --- |
| No | **mAb** | **Conj** | **Clone** | **Cat#** | **Supplier** |
| 1 | CD3 | PE | HIT3a | 300308 | Biolegend |
| 2 | CD3 | APC | OKT3 | 317317 | Biolegend |
| 3 | CD8a | PE-Cy5 | HIT8a | 300909 | Biolegend |
| 4 | CD8a | FITC | RTA-T8 | 302006 | Biolegend |
| 5 | CD8 | Alexa Flour-700 | SK1 | 344724 | Biolegend |
| 6 | CD4 | PerCP | OKT4 | 317431 | Biolegend |
| 7 | CD4 | APC | A161A1 | 357408 | Biolegend |
| 8 | CD25 | PE | BC96 | 302605 | Biolegend |
| 9 | CD62L | APC/Cyanine7 | DREG-56 | 304813 | Biolegend |
| 10 | CD62L | Violet 610 | DREG-56 | 304834 | Biolegend |
| 11 | CCR7 | PE/Cyanine7 | G043H7 | 353225 | Biolegend |
| 12 | CD45RO | APC | UCHL1 | 304210 | Biolegend |
| 13 | CD45RA | APC | HI100 | 304112 | Biolegend |
| 14 | CD54 | APC/Fire 750 | HA58 | 353122 | Biolegend |
| 15 | CD56 | PE/Cyanine 7 | 5.1 H11 | 352510 | eBioscience |
| 16 | NKG2D | APC | 1D11 | 562064 | BD Pharmingen |
| 17 | TCR β chain | APC | H57-597 | 109212 | Biolegend |
| 18 | TCR vβ13.1 | FITC | H131 | 312403 | Biolegend |

**
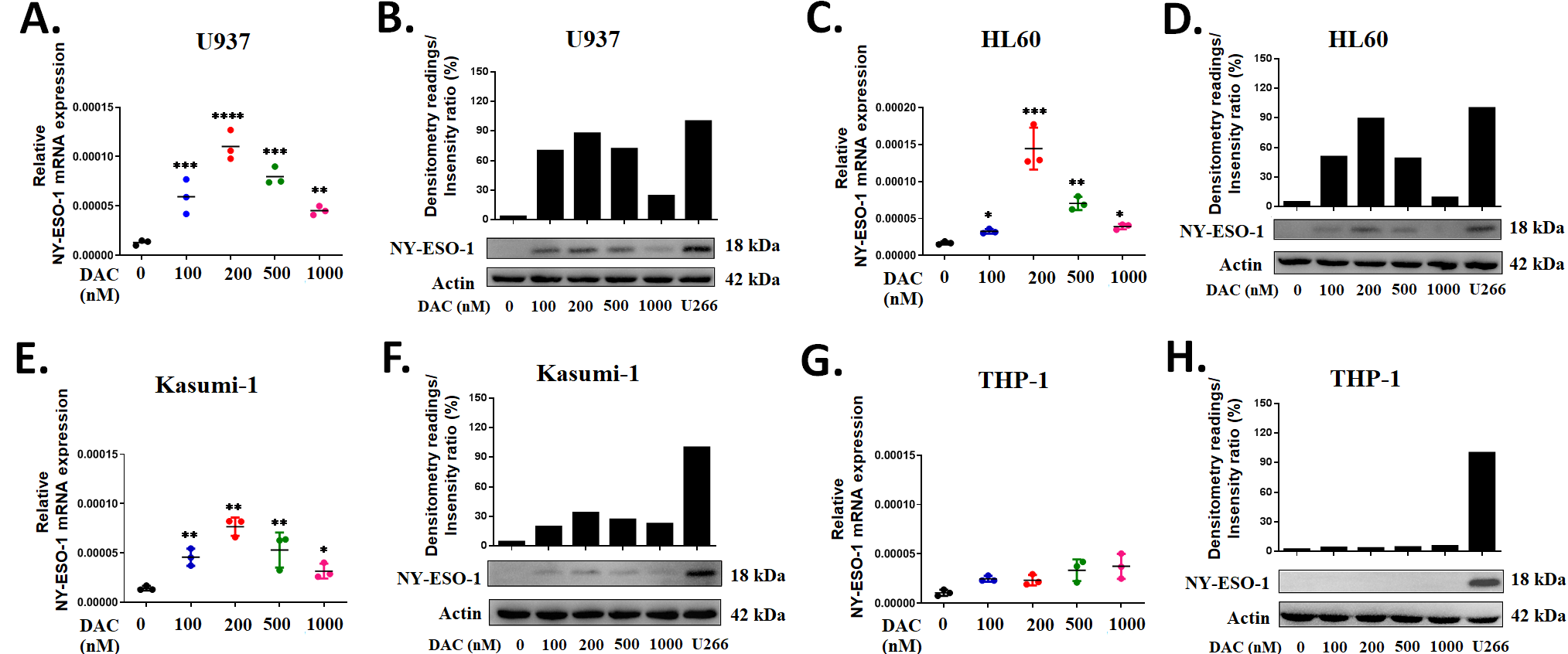
Supplementary figures:**

**Supplementary Figure S1.** **Decitabine induces NY-ESO-1 expression in AML cell lines in a dose-dependent manner**. qRT-PCR analysis of NY-ESO-1 mRNA expression in U937, HL60, Kasumi-1, and THP-1 cells receiving serial doses (100 nM, 200 nM, 500 nM, and 1000 nM) of DAC for 72 h. (**A**, **C**, **E**, **G**). The mRNA levels were determined by normalizing *Ct* value of NY-ESO-1 to β-actin, and qualified by using the method (2^-ΔΔCt^). The data are presented as the mean ± sd of three representative tests (*n* = 3). Statistical comparisons between two groups were determined by two-tailed unpaired t tests. **P* < 0.05; ***P* < 0.01; ****P* < 0.001; *****P* < 0.0001). (**B, D, F, H**) Western blot analysis of NY-ESO-1 protein levels and densitometry reading/intensity ratios in the four AML cell lines. Membranes were incubated with anti-NY-ESO-1 antibody (Clone SP349; Abcam) and β-actin (Clone 13E5; Cell Signaling) as a loading control. Multiple myeloma U266 was used as a control for NY-ESO-1 protein expression.


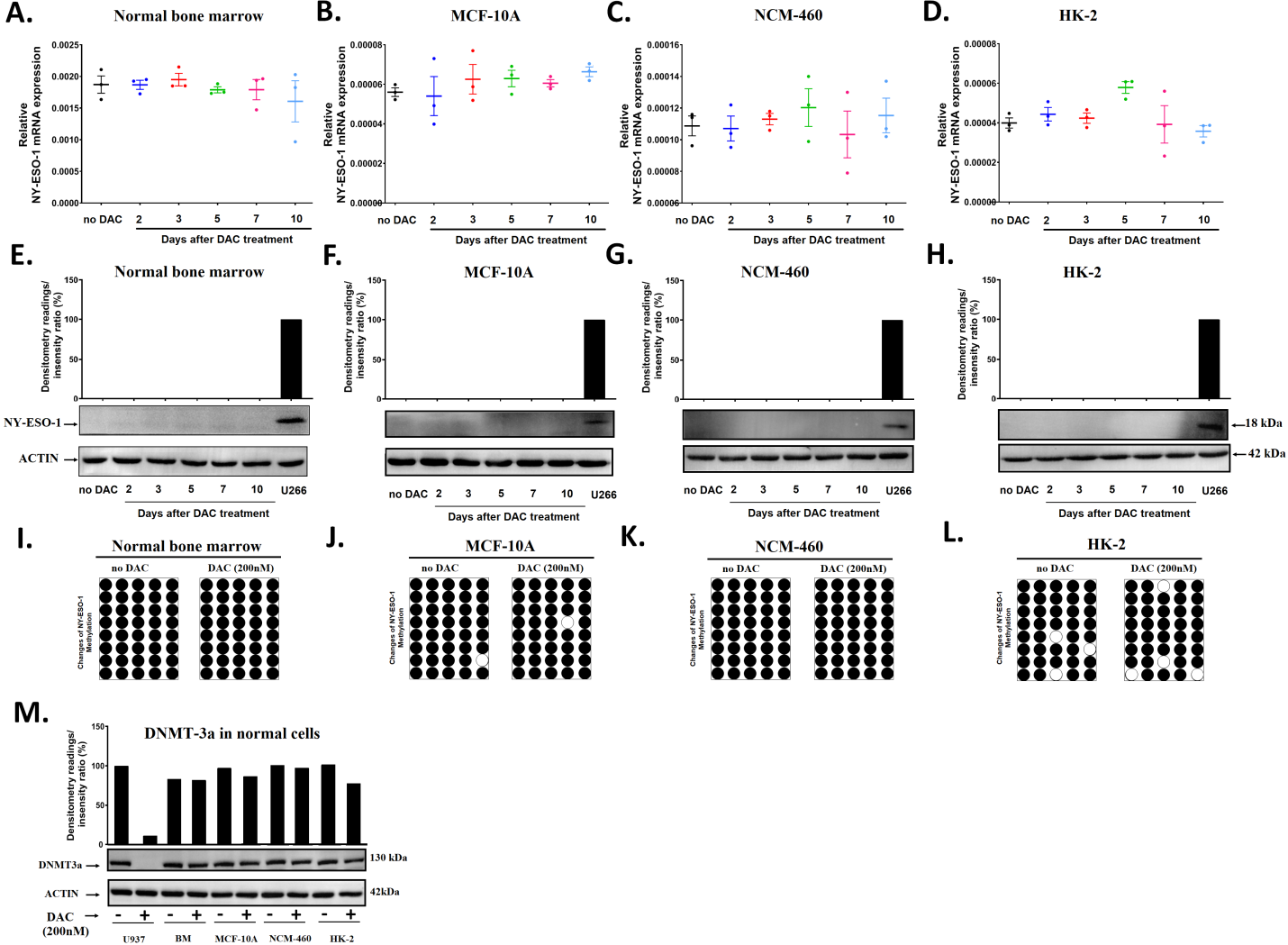


**Supplementary Figure S2. NY-ESO-1 expression in normal cells is not upregulated by decitabine**. **(A-D)** qRT-PCR analysis of NY-ESO-1 mRNA levels in normal human bone marrows and human normal cell lines (breast epithelial cell, MCF10A; colon epithelia cell line, NCM460; kidney proximal tubular 2, HK-2) in 10 days after treatment with 200 nM DAC. **(E- H)** Western blot analysis of NY-ESO-1 levels in normal bone marrow, human normal cell lines (MCF10A, NCM460, HK-2), and multiple myeloma cell line U266 as positive control. **(I- L)** Bisulfite sequencing analysis of methylation status of NY-ESO-1 promoters in normal bone marrow and human normal cell lines (MCF10A, NCM460, HK-2) that treated with 200 nM of DAC for 72 h (*n* = 8). **(M)** Western blot analysis of DNMT3a levels in normal bone marrows and human normal cell lines (MCF10A, NCM460, HK-2) in 3 days after treatment with 200 nM DAC for 72 h.


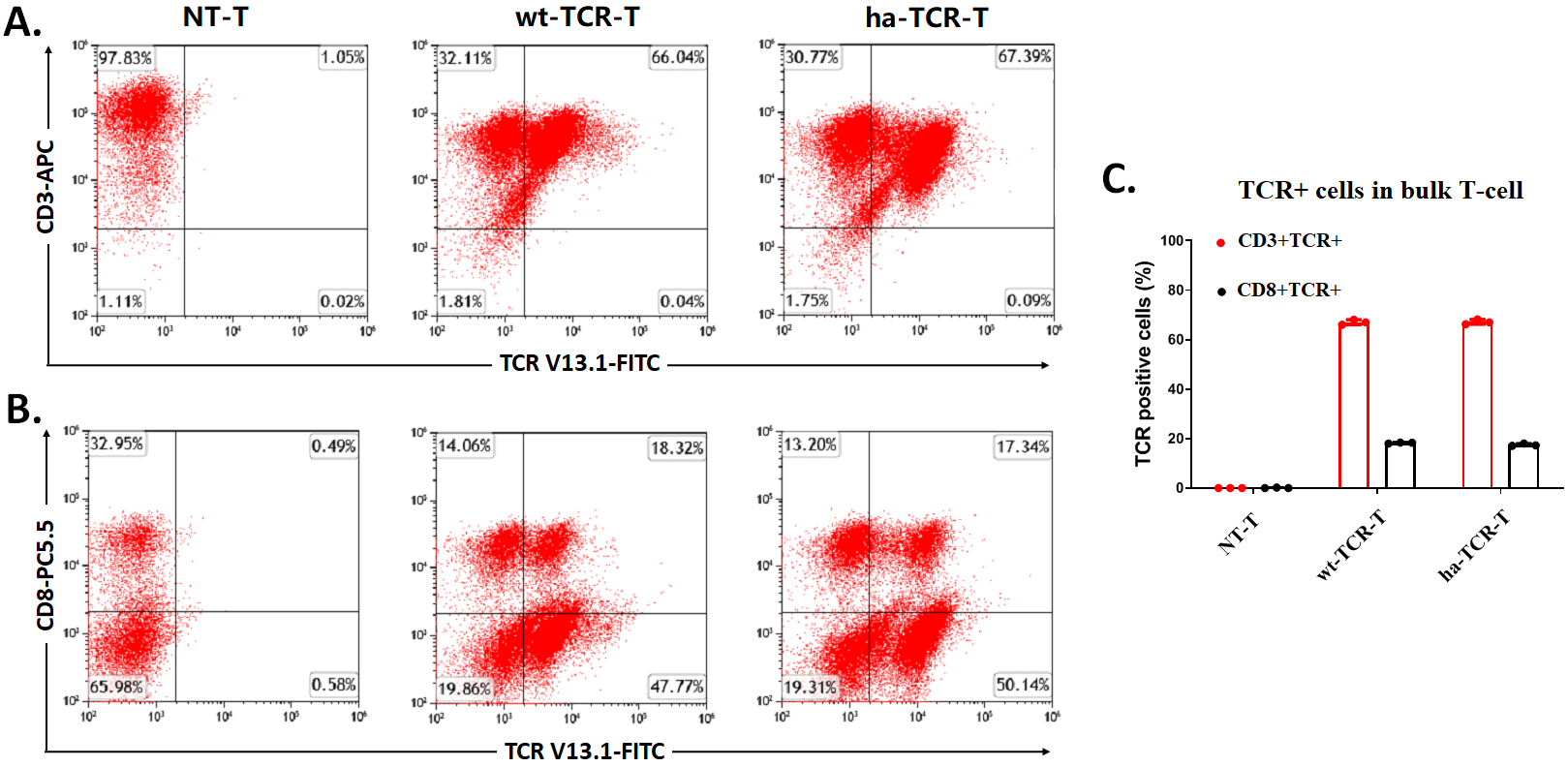


**Supplementary Figure S3. Flow cytometry detection of NY-ESO-1 specific TCR-T cells**. Untransduced T (NT-T) cells, wild type TCR-T (wt-TCR-T) cells, and high-affinity TCR-T (ha-TCR-T) cells were co-stained with conjugated monoclonal antibody of anti-human TCR vβ13.1 with **(A)** CD3 and **(B)** CD8. **(C)** The bar chart represents co-expression of TCR+ cells in bulk T-cell from A and B (n = 3).


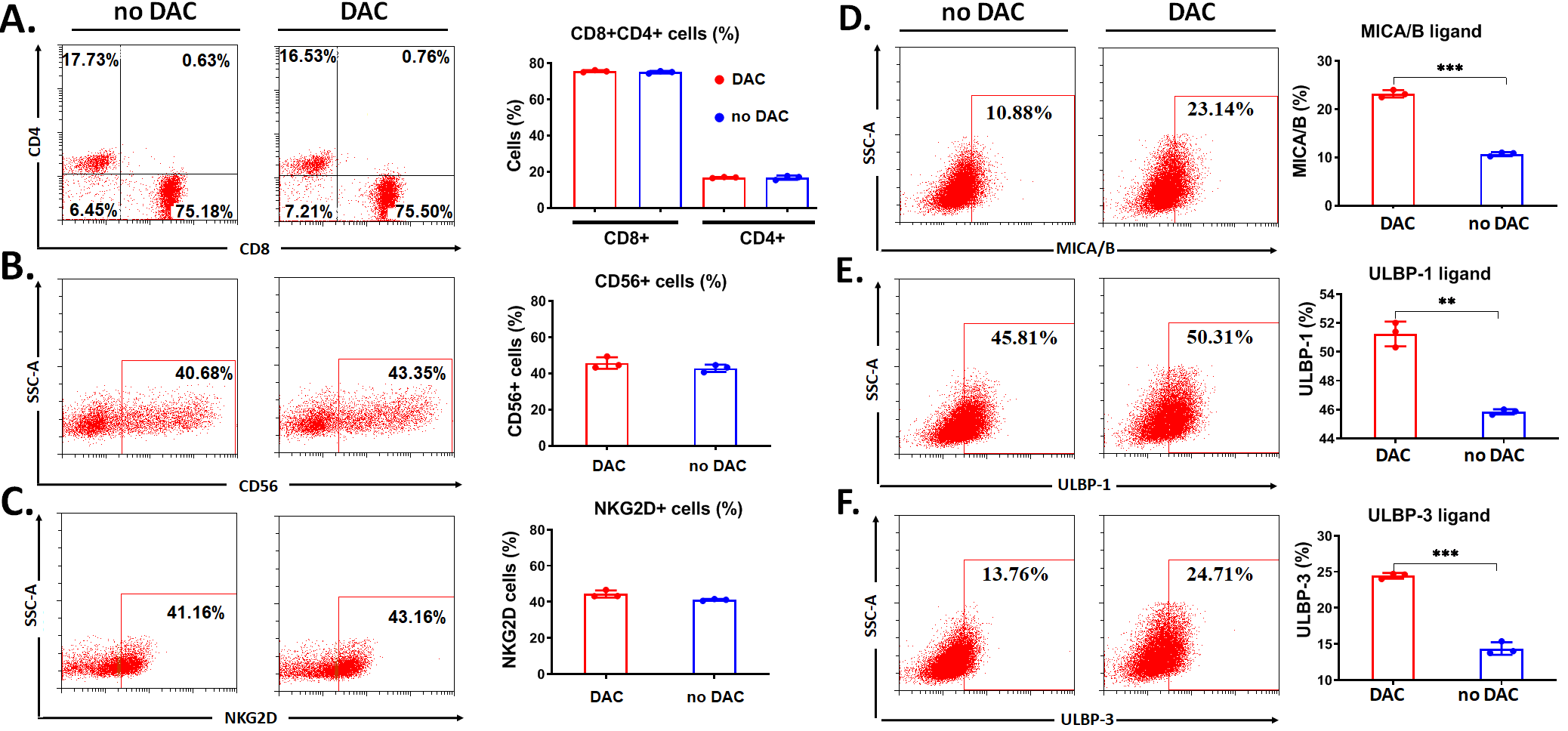


**Supplementary Figure S4. Flow cytometry detection of NKG2D receptors on NT-T cells and NKG2D ligands in AML cells**. NT-T cells were co-cultured with DAC-induced U937-A2^+^ cells for 20 h. The U937-A2^+^ cells were induced with 200 nM DAC for 72 h. **(A)** Proportion of cytotoxicity (CD8^+^ and CD4^+^) cells, **(B)** nature killer cells (CD56^+^), and **(C)** cells expressing activating NKG2D receptors. Increased expressions NKG2D ligands were detected in DAC-treated U937 cells treated with 200 nM DAC for 72 h. Phenotypic analysis of NKG2D ligands **(D)** MICA/B, **(E)** ULBP-1 and **(F)** ULBP-3 in U937-A2^+^ cells with and without DAC treatment. The data are presented as the mean ± sd (n = 3). Two-tailed unpaired t test was applied to compare NKG2D ligands expression between DAC and noDAC groups. ***P* < 0.01; ****P* < 0.001.

**
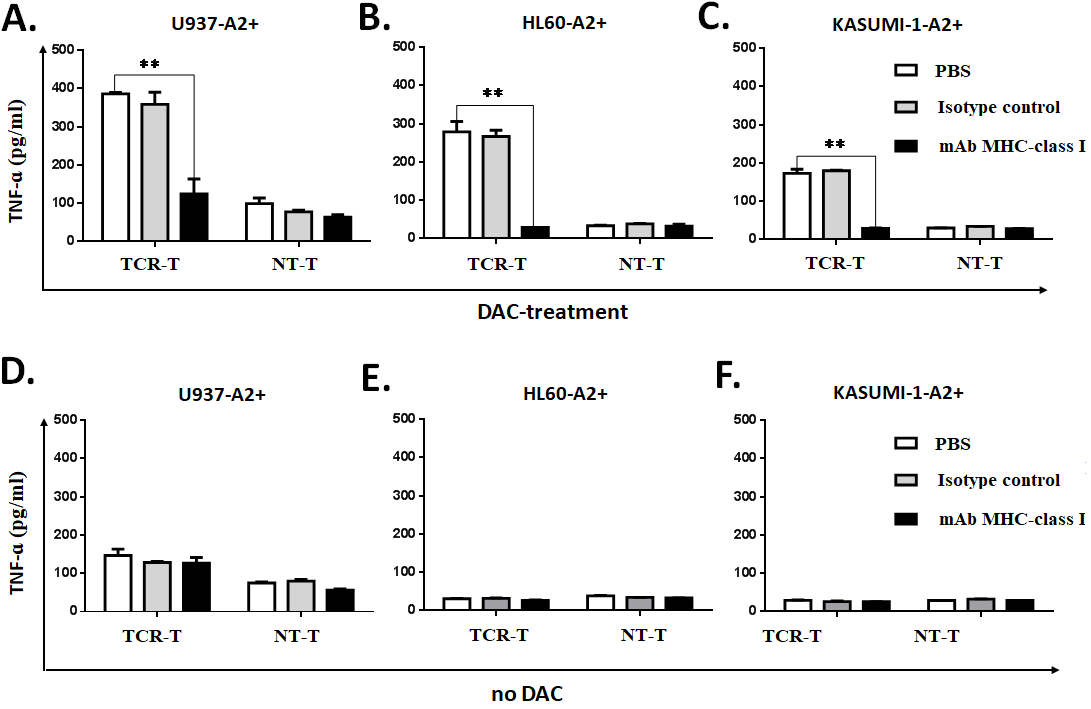
**

**Supplementary Figure S5.** **Anti-leukemia activities of NY-ESO-1 specific TCR-T cells are MHC-dependent**. For each cell (U937-A2^+^, HL60-A2^+^, and Kasumi-1-A2^+^), a total of 2×10^4^ cells were treated with 200 nM DAC for 72 h. **(A-C)** DAC-treated and **(D-F)** untreated cells were loaded with mAb MHC-class I monoclonal antibody (W6/32, Biolegend) or with isotype control (Ig2a isotype; Clone MOPC-173, Biolegend) with a final concentration of 10 µg/mL, followed by co-culture with effector cells at an effector-to-target ratio of 5:1 ratio for 20 h. Data shown are mean ± sd of three representative tests (n = 3). Statistical comparisons between two groups were determined by two-tailed unpaired t-test. ***P* < 0.01.


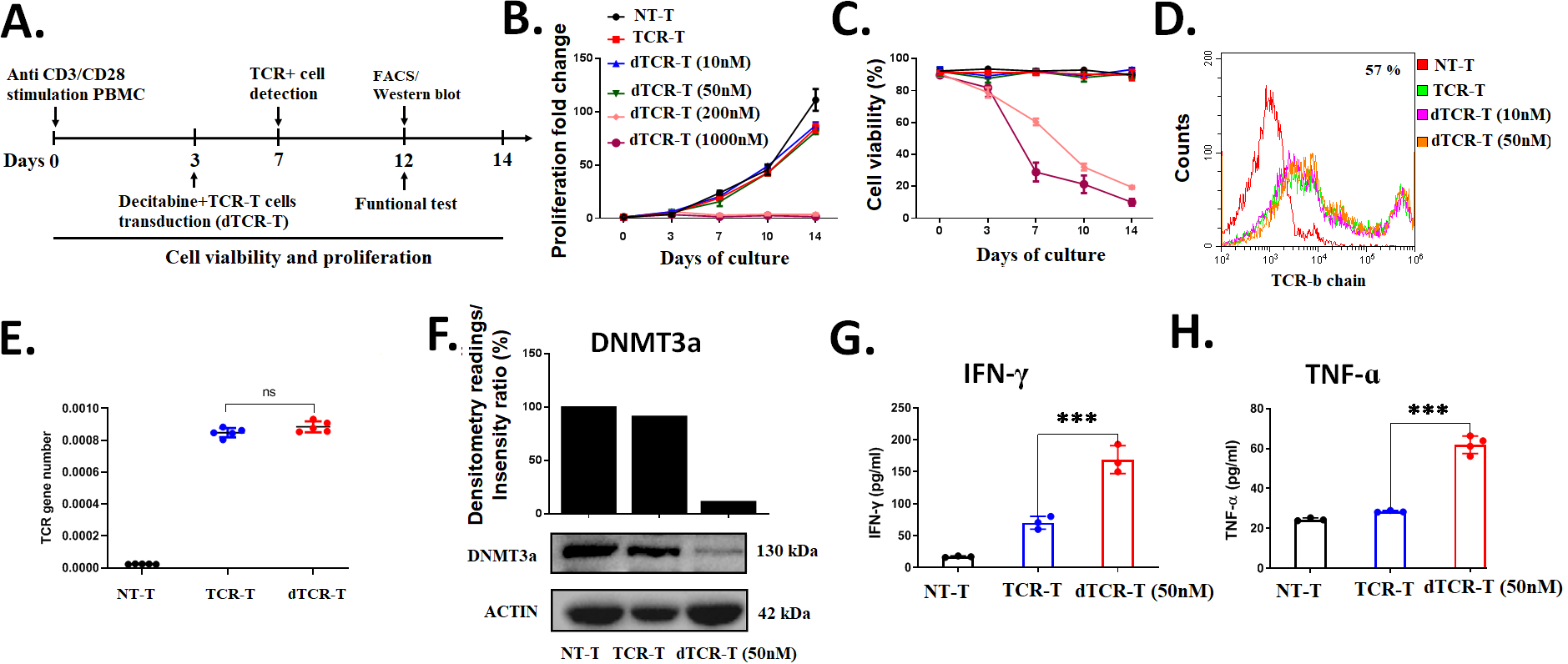


**Supplementary Figure S6. The effect of decitabine in dTCR-T transduction and function. (A)** Schematic representation of generating dTCR-T cells. DAC was added at the same time as the TCR-T gene transduction and removed out after 24 h. (**B)** Proliferation and **(C)** viability of TCR-T cells in response to various doses of DAC (10 nM, 50 nM, 200 nM, and 1000 nM). **(D)** Flow cytometry analysis of the percentages of TCR-positive cells in NT-T, TCR-T, and dTCR-T cells (transduced with 10 nM and 50 nM). **(E)** The TCR gene number in NT-T, TCR-T, and dTCR-T (50 nM DAC) from cell expansion was measured by qRT-PCR (*n* = 5). **(F)** Western blot detection of the DNMT3a protein expression levels and densitometry analysis of the ratio of DNMT3a/β-actin protein expression levels in NT-T, TCR-T, and dTCR-T cells (transduced with 50 nM of DAC; *n* = 3). Increased secretions of **(G)** IFN-γ and **(H)** TNF-ɑ by dTCR-T cells were analyzed by ELISA on day 12 after co-culture with target cells. NT-T, TCR-T, and dTCR-T cells were cultured in a fresh medium without IL-2 for 20 h (*n* = 3). The data in **B**, **C**, **D**, **E**, **G** and **H** shown are mean ± sd. Statistical comparisons between two groups in **E, G,** and **G** were determined by two-tailed unpaired t tests. ****P* < 0.001; *ns* not significant.

**
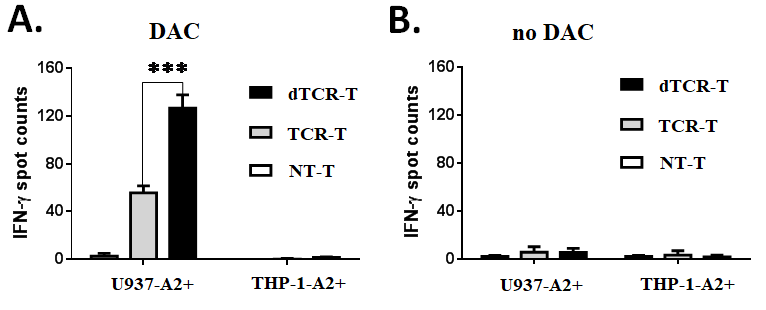
**

**Supplementary Figure S7.** **Decitabine empower dTCR-T cells with enhanced function against AML cells**. ELISpot analysis of IFN-γ production by T cells after stimulation with **(A)** DAC-treated or **(B)** untreated target AML cells. 2×10^5^ target cells (U937-A2^+^ and THP-1-A2^+^) were treated with 200 nM DAC for 72 h, and co-cultured with NT-T, TCR-T, or dTCR (transduced with 50 nM of DAC) at an effecter-to-target of 1:20 for 20 h. Statistical comparisons between two groups were determined by two-tailed unpaired t tests. ****P* < 0.001.


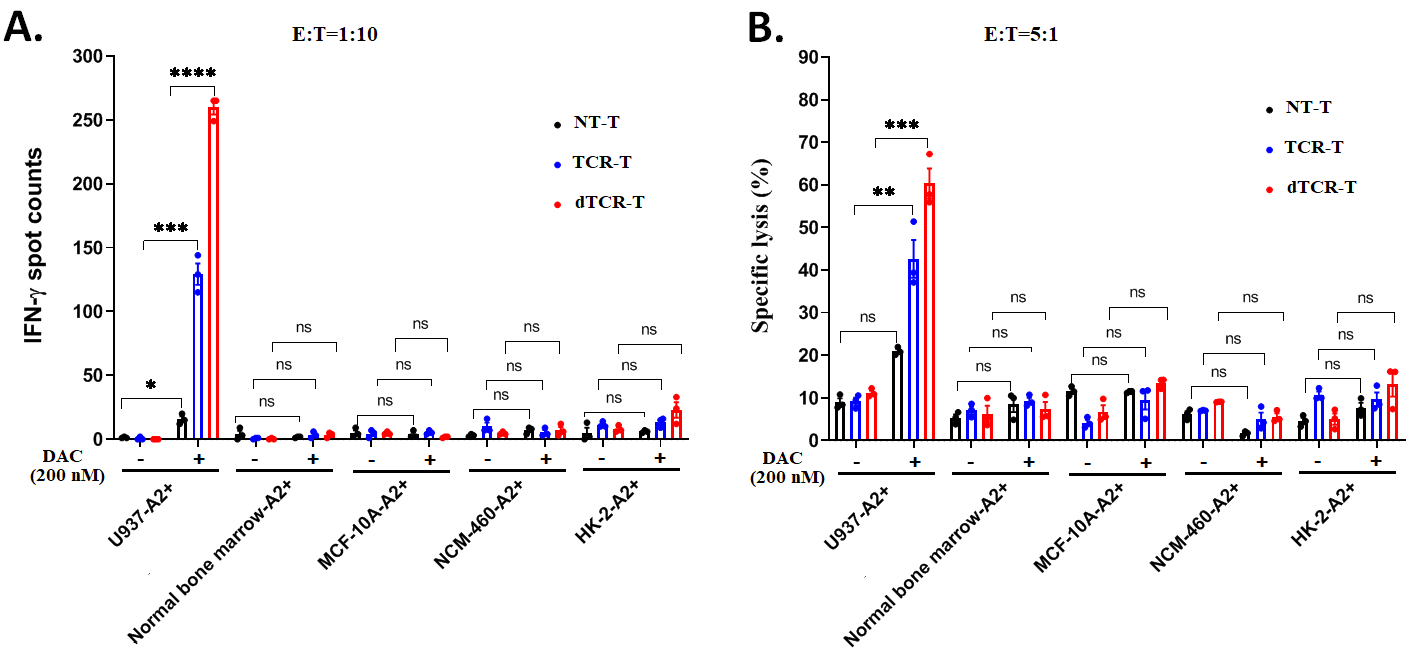


**Supplementary Figure S8. High-affinity NY-ESO-1-specific TCR-T cells show no toxicity to normal human cells. Normal human bone marrow cells (HLA-A2^+^), human** normal cell lines (breast epithelial cell, MCF10A-A2^+^; colon epithelia cell line, NCM460-A2^+^; kidney proximal tubular 2, HK-2-A2^+^)**, and AML cell line U937-A2^+^ (used as positive control) were treated with 200 nM DAC for 72 h as described in the materials and methods. (A)** ELISpot analysis of IFN-γ 1×10^3^ NT-T, TCR-T, or dTCR-T cells that stimulated by untreated or DAC-treated target cells at an effector-to-target (E:T) ratio of 1:10 for 20 h. **(B)** Specific lysis of 2×10^4^ target cells was measured by LDH-release assay at E:T =5:1 ratio. The data in A-B are presented as the mean ± sd (*n*=3). Statistical comparisons between two groups were determined by two-tailed unpaired t tests. **P <* 0.05; ***P <* 0.01; ****P <* 0.001; *****P <* 0.0001; *ns* not significant.
